# S100B and NSE serum concentrations after simulated diving in rats

**DOI:** 10.14814/phy2.12546

**Published:** 2015-10-13

**Authors:** Marianne B Havnes, Yvonne Kerlefsen, Andreas Møllerløkken

**Affiliations:** Department of Circulation and Medical Imaging, Norwegian University of Science and TechnologyTrondheim, N-7489, Norway

**Keywords:** Biomarker, blood–brain barrier, central nervous system, diving

## Abstract

The purpose of this study was to assess whether one could detect S100 calcium-binding protein B (S100B) and neuron-specific enolase (NSE) in serum of rats after a simulated dive breathing air, with the main hypothesis that the serum concentrations of S100B and NSE in rats will increase above pre-exposure levels following severe decompression stress measured as venous gas emboli (VGE). The dive group was exposed to a simulated air dive to 700 kPa for 45 min. Pulmonary artery was monitored for vascular gas bubbles by ultrasound. Pre- and postdive blood samples were analyzed for S100B and NSE using commercially available Elisa kits. There was no increase in serum S100B or NSE after simulated diving and few of the animals were showing high bubble grades after the dives. The present study examined whether the protein biomarkers S100B and NSE could be found in serum from rats after exposure to a simulated dive to 700 kPa for 45 min breathing air. There were no differences in serum concentrations before versus after the dive exposure. This may be explained by the lack of vascular gas bubbles after the dives.

## Introduction

Diving is associated with multiple risks, and for any diver venturing beneath the surface of the sea, the change of pressure is undoubtedly the most important factor to consider. Decompression sickness (DCS), a pathological condition due to bubble formation from dissolved inert gases during or following decompression, is one of the greatest threat to any diver (Francis and Mitchell 2003a).

Although the overall incidence of diving-related DCS is relatively small, with a prevalence of 2.0 per 10,000 dives (Pollock et al. 2008), it is a serious condition with a lethal outcome for some. Among recreational divers, neurological symptoms have in two larger epidemiological studies been found to be the most common manifestations. Of approximately 3500 cases compiled from a DAN study, over 40% where neurologic, and in the 1170 cases recorded at the Institute of Naval Medicine (INM) in the United Kingdom, more than 77% of the cases had neurological manifestations (Francis and Mitchell 2003b). Acute DCS affecting the brain usually results in serious neurological dysfunction, and multiple instances have been linked to long-term damage (Daniels 2003). When neurological damage occurs in divers, the suspected primary cause is vascular gas bubbles. Acute effects can be caused by extravascular bubbles producing pain, but vascular bubbles may grow in size and cause infarction, inducing stroke-like symptoms (Vann et al. 2011). Bubbles can reach the brain through the arterial circulation, either through a right-to-left shunt in the heart, or they may overwhelm the pulmonary system and pass into the arteries (Warren et al. 1988).

In addition to the pathogenic effects of cerebral bubbles after diving, some subclinical changes are proposed. For example, a change in blood–brain barrier (BBB) permeability is found in animals performing simulated dives (Nohara and Yusa 1997; Hjelde et al. 2002a). Decompression has been shown by histological staining to cause increased permeability of the BBB and with size of permeability correlated to amount of visible intravascular bubbles (Chryssanthou et al. 1977).

The biomarkers, (S100 calcium-binding protein B) S100B and (Neuron-specific enolase) NSE, are thought to be released into the blood as a consequence of an impaired BBB, or brain injury due to tissue ischemia or edema (Yardan et al. 2009), and increased serum concentrations have been seen following cerebrovascular disease (Schaarschmidt et al. 1994; Herrmann et al. 2000), as well as in numerous other central nervous system (CNS) disorders (Kaiser et al. 1989; Rothermundt et al. 2003).

The development in ultrasonic imaging and Doppler technology has allowed for the detection of (Venous gas emboli) VGE that are recognized as a valid measure of decompression stress (Nishi 1990; Brubakk and Mollerlokken 2009). Even though the presence of VGE is statistically correlated with the risk of DCS (Sawatzky 1991), the relationship between gas bubbles and DCS is not always straightforward. Studies (Bayne et al. 1985) have reported incidents of DCS without the presence of gas bubbles, and conversely, gas bubbles have been detected in asymptomatic divers, so called “silent bubbles” (Nishi 1990). This seemingly discrepancy has raised the discussion if detection bubbles after diving have any scientific and medical value, and have led to the investigation of possible other biomarkers to support investigations of divers post dive in order to address whether they have undertaken a stressful dive or not. From investigations of traumatic brain injury we know that a number of biomarkers are used, among these are S100B and NSE (Zurek and Fedora 2012). Serum levels of S100B have been shown to be increased in goats and rats after deep dives with rapid decompression (Havnes et al. 2010; Jurd et al. 2001). Symptoms of brain injury after DCS are often mild and not disabling. However, some individuals suffer for months and years from headaches, memory changes, poor concentration, and other neurological symptoms (Dutka 2003). They also resemble the symptoms of traumatic brain injury (Nolan 2005; Dutka 2003). Knowledge of brain sequelae during and after a dive is still lacking, both in terms of short- and long-term effects.

The aim of the present study was to assess whether one could detect S100B and NSE in serum of rats after simulated dives breathing air, with the main hypothesis that the serum concentrations of S100B and NSE in rats will increase above pre-exposure levels following severe decompression stress measured as VGE.

## Methods

### Experimental animals

A total of 26 female (280.15 ± 12.67 g, 9–10 weeks old) Sprague Dawley rats (Taconic M&B, Denmark) were included in the study. The rats were divided into a test (*n *=* *16) and control group (*n *=* *10). The animals were housed in individually ventilated cages in an accredited animal care facility, with free access to food and water. Acclimation was set to 1 week. Light was controlled on a 12-h dark/light cycle, with light periods between 7 am and 7 pm. Temperature was 19–22°C and humidity 50–60%. The animals were transferred to the diving laboratory 24 h prior to the simulated dive. The experimental procedure conformed to the European Convention for the Protection of Vertebrate Animals used for Experimental and other Scientific Purposes. Before commencing, the Norwegian Council for animal research reviewed and approved all aspects of the study.

### Procedure and simulated dive protocol

Blood samples for analysis of S100B and NSE were collected from all animals at three different time intervals: 2 weeks prior to the simulated dive, in order to establish pre-exposure serum concentrations, and 1 and 48 h after the dive. All animals received the same treatment, except for the simulated dive, which only the test group was exposed to.

Prior to the first blood sampling, the rats were weighed and anesthetized with a haldol mixture containing haldol (5 mg/mL, Jansen-Cilag), fentanyl (0.05 mg/mL,. Alpharma), and midazolam (5 mg/mL Alpharma). About 0.2 mL per 100 g of body weight was injected subcutaneously into the lower back and 1 mL of blood was drawn from the saphenous vein by puncturing it with a 23G × 1″ syringe, directly collecting the blood into Eppendorf tubes.

The dive profile was selected based on previous studies on rats with comparable body weight (Havnes et al. 2010). The animals in the test group, 3 or 4 at a time, were placed in a 20-L dry hyperbaric chamber and were subjected to a simulated dive to a pressure of 700 kPa while breathing air. The compression rate was 200 kPa per min and bottom time was 45 min. At the end of the exposure period, the animals were decompressed linearly to the surface (100 kPa) at a rate of 50 kPa per min. To remove CO_2_ from the chamber atmosphere, soda lime was put on a tray below the rats. Immediately after surfacing, the animals were anesthetized (0.4 mL/100 g), and the pulmonary artery was monitored for VGE using a 10 MHz transducer connected to a system FiVe ultrasound scanner (GE Vingmed Ultrasound AS, Norway). The animals were monitored for VGE at intervals of 15, 30, 45, and 60 min after surfacing. Breathing patterns was observed and temperature and survival times were recorded. One hour after surfacing the second blood sample was taken from both test animals and controls, using the same method as described earlier. The animals were then put back into their cages where they had free access to water and a pellet rodent diet. The final blood sample was drawn 48 h after the dive.

All blood samples were allowed to clot for 30 min at room temperature and centrifuged (Eppendorf Centrifuge 5810 R) at 3200 rpm for 10 min. The serum was stored frozen at −80°C for approximately 1 month before assay analyses were performed by commercially available Elisa Kits (BioVendor-Laboratorni medicina, Czech Republic, S100B Elisa kit [cat. no. RD192090100R], UScn Life Science Inc., P. R. China, NSE Elisa kit [cat. no. E0537Ra]). Due to the number of samples, two kits were used for each biomarker.

### Bubble detection

Bubbles were seen as bright streaks on the monitor screen, and were also verified by Doppler. The bubbles were graded on a scale from 0 to 5 according to a previously described method by Eftedal and Brubakk (1997), where bubble grade 0 equals no bubbles, 1 equals an occasional bubble, 2 equals at least one bubble/4th heart cycle, 3 equals at least one bubble/heart cycle, 4 equals continuous bubbling, and 5 equals massive bubbling also described as “white-out” as individual bubbles cannot be seen. Bubble grade 2 and lower is considered a low bubble grade with a relatively low risk of developing DCS, while a bubble grade higher than 2 is considered a high bubble grade (Eftedal and Brubakk 1997).

### Statistical analysis

The statistical analysis was performed in R (R Development Core Team, 2009) by linear mixed-effects modeling, using the lme4 package (Bates and Maechler 2010). The models included either NSE or S100B as the response variable, and identical fixed and random effects. There were multiple measurements per rat, and consequently the measurements were not independent. Thus, all models were fitted with random effects terms to account for temporal dependence due to pseudoreplication. Furthermore, 3–4 rats were selected for decompression at a time, and consequently, a random effect term was also fitted to account for any variation among dive groups.

The fixed effects, or predictor variables, were time of measurement and a control/test group factor. Time of measurement was treated as a categorical predictor variable as one measurement was taken before and two were taken after the simulated dive. The control/test group factor was simply a factor with two levels, where one level included rats that were subjected to the simulated dive, and the other, rats that were not. The control and test individuals were treated identically prior to the simulated dive. Thus, no significant difference between the control and test individuals in the first measurement, and a significant difference in either the second or the third measurement, or both, would indicate a positive result. A negative result would be no significant difference between the test and the control group in any measurement. To test this, we fitted a model with an interaction between time of measurement and the control/test group variable, and a model with no predictor variables. The models have been named the full and null model, respectively. Thus, in the null model, there is only a grand mean and any variation in the response is attributable to random variation among dive groups and rats, while in the full model, much of this variation is attributable to the predictor variables.

## Results

### Biomarker analyses

#### S100B

**Figure 1:**
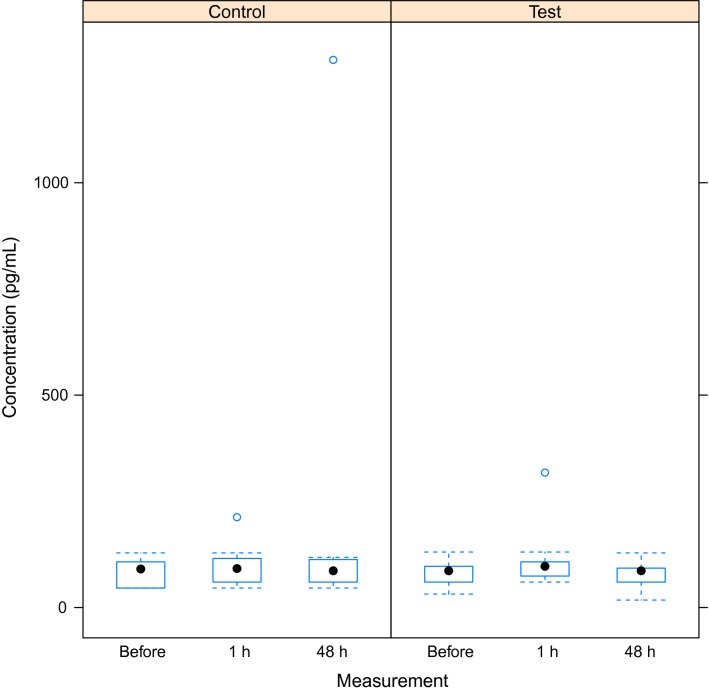
Box plot of S100B concentration (pg/mL) for the test and the control group at measurement 2 weeks before, and 1 and 48 h after the simulated dive, respectively. Median, 25th and 75th percentile are shown. Vertical lines represent the largest and smallest values except for extreme outliers presented as circles.

There was no increase in serum concentrations of S100B in the test group after exposure to the simulated dive (Fig.1). The best model was the null model (Table1). However, there was increasing variation from the first to the last measurement, but this increase is attributable to a few extreme measurements in both the test and control group. Thus, our results indicate that any variation in the concentration of S100B is attributable to random variation among rats and dive groups (S100Bconc. = 86 ± 7 pg/mL), and not to the simulated dive.

**Table 1:** Model selection by AICc for S100B

Model	K	AICc	∆AICc	ModelLik	AICcWt	LL	Cum.Wt
Null	10	847.56	0.00	1.00	0.99	−412.00	0.99
Full	15	856.09	8.54	0.01	0.01	−408.84	1.00

Both models included random effect terms to account for any temporal dependence due to pseudoreplication and variation among dive groups. The full model included a fixed effect term for the interaction between time of measurement and control/test group variables. The null model included no predictor variables. The reported values are the number of parameters in a given model (K), the difference in AICc value relative to the minimum value of the candidate set (∆AICc), the probability of being the best among the candidate models (AICcWt), the cumulative probability (Cum.Wt), and the log likelihood (LL).

#### NSE

**Figure 2:**
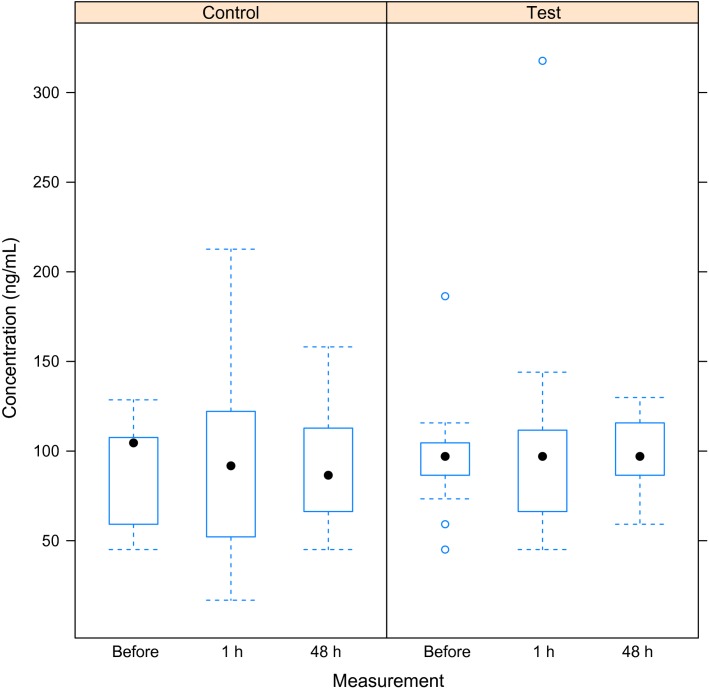
Box plot of NSE concentration (pg/mL) for the test and the control group at measurement 2 weeks before, and 1 and 48 h after the simulated dive, respectively. Median, 25th and 75th percentile are shown. Vertical lines represent the largest and smallest values except for extreme outliers presented as circles.

There was no increase in serum concentrations of NSE in the test group after exposure to the simulated dive (Fig.2). The best model was the null model (Table2).

**Table 2:** Model selection by AICc for NSE

Model	K	AICc	∆AICc	ModelLik	AICcWt	LL	Cum.Wt
Null	10	749.38	0.00	1.00	1.00	−362.91	1.00
Full	15	763.03	13.65	0.00	0.00	−362.30	1.00

Both models included random effect terms to account for any temporal dependence due to pseudoreplication and variation among dive groups. The full model included a fixed effect term for the interaction between time of measurement and control/test group variables. The null model included no predictor variables. The reported values are the number of parameters in a given model (K), the difference in AICc value relative to the minimum value of the candidate set (∆AICc), the probability of being the best among the candidate models (AICcWt), the cumulative probability (Cum.Wt), and the log likelihood (LL).

### Bubble detection

All together, the simulated dive protocol resulted in very few bubbles (Table3). Of the 16 rats in the test group, only seven rats had bubbles. Six rats were graded with bubble grade 1, and one rat was graded with bubble grade 5. This rat showed signs of paralysis and died 5 min after decompression. All blood vessels were filled with gas, and gas was also present in the adipose tissue.

**Table 3:** Bubble grade observed in test group after simulated dive to 700 kPa

	Bubble grade 1–2	Bubble grade >2
Rats (*n*)	6	1

Showing number of individuals with detectable bubbles divided into two groups; bubble grade 1–2 and bubble grade above 2.

## Discussion

The present study show no blood–brain leakage on serum biomarker analyses after simulated dive in rat. Very few of the animals were showing high bubble grades after the dives. This is in contrast to what we have seen previously in rats following the same decompression profile.

A number of studies have reported a correlation between serum levels of S100B and NSE and brain damage. Following acute stroke, a condition in which the clinical manifestations are similar to neurological DCS, it has been found that S100B blood concentrations were correlated with infarct volume and neurological outcome (Herrmann et al. 2000). Also, in a review on S100B as a biochemical marker of brain injury, it was concluded that serum S100B was an accurate measure for assessing severity and neurological outcome in patients diagnosed with cerebral stroke or ischemia (Korfias et al. 2006).

Nonetheless, when reviewing the literature, there have been some concerns regarding S100B as a marker of brain damage. Increased levels of S100B have been found following trauma and cardiac surgery without any associated brain injury (Pelinka and Boltzmann 2005). It has also been discovered that restraint stress can increase S100B levels in rats (Scaccianoce et al. 2004). Hence, the presence of S100B in serum is not strictly limited to brain damage.

With regard to NSE and decompression stress, there are no previous studies that the present results can be compared to but previous studies on neurological conditions similar to neurological DCS have showed promising results. Schaarschmidt et al. (1994) studied the clinical relevance of blood concentrations of NSE in patients with cerebrovascular diseases (Schaarschmidt et al. 1994). Plasma levels of NSE were measured in patients with brain infarction, intracerebral hemorrhage, and ischemia during the first 10 days after the acute event. It was found that in cases of ischemia there was a close correlation between plasma NSE values during the first 72 h and clinical outcome, suggesting that NSE correlates with the degree of neuronal damage. Also, in a review by Anand and Stead (2005), it was concluded that serum levels of NSE were higher in stroke patients than in controls, and that levels of NSE also correlated with the volume of infarcted tissue (Anand and Stead 2005). This supports the conclusion that in the present study, the applied dive probably caused little or no brain damage.

It has been recognized for over a century that neurological damage may arise as a consequence of severe decompression stress, and hence, several attempts have been made to use potential biomarkers to address adverse effects of diving on the central nervous system. In a study by Havnes et al. (2010), levels of S100B were increased following a dive to 700 kPa for 45 min breathing air compared to control animals that did not undergo the simulated dive. In that study, there was a trend toward levels of S100B being correlated with the amount of vascular gas bubbles detected after the dive (Havnes et al. 2010).

The connection between vascular gas bubbles and adverse effects of decompression is not a straightforward and linear relationship, but studies have shown that the absence of VGE correlates well with the absence of DCS (Sawatzky 1991; Nishi et al. 2003). It is recognized that divers commonly develop VGE on decompression, and that the bubbles are formed from super-saturated gases in the tissues or blood upon decompression (Tikuisis and Gerth 2003). Large individual and intraindividual differences exists, however, and it has been reported large quantities of VGE in divers without any clinical signs or symptoms of DCS.

The pathological effects of bubbles may cause a mechanical disruption of the tissue concerned, with the compression of noncompliant tissue of blood vessels and lymphatics, or from simply obstructing blood vessels (Nossum et al. 1999). Several studies have shown reduced endothelial function after exposure to vascular bubbles (Nossum et al. 1999; Hjelde et al. 2002b; Brubakk et al. 2005; Lambrechts et al. 2013) and we have suggested that this may be a central mechanism in the development of serious decompression injury (Brubakk and Mollerlokken 2009). Albeit the link between VGE and DCS is far from clear, the assumption was made that a large bubble load would lead to the arterialization of VGE, which in turn would lead to neurological DCS, and subsequent neurological damage or BBB impairment.

Poff et al. (2007) failed to show any increase in S100B levels following acute DCS. It must be stressed, however, that this study had several methodological concerns, including selection bias of the patients, and a lack of a control group (Poff et al. 2007). Additionally, diagnosis was delayed for an average of 3 days, and considering the short half-life of S100B (30 min), the biomarker may have been at undetectable concentrations at the time of measurement (Ghanem et al. 2001).

Previous findings from our laboratory indicate that when rats weighing around the weight we used in the present study, we should get a lot of bubbles formed after this dive. In the present study, however, this dive profile produced very few bubbles, and only one animal experienced a massive bubble load (grade 5), and had observable signs of neurological DCS. In comparison, 8 of the 11 animals in the study by Havnes et al. (2010) showed a bubble grade of 5. Thus, given this assumption it is unlikely that a bubble grade of 1 would lead to neurological damage, and this could explain the stable levels of S100B and NSE found in the present study.

It is possible that the presence of bubbles may have failed to be detected in the pulmonary artery, but this it is unlikely as an experienced observer was present at all times. The process of bubble formation is complex and largely unknown, and it is therefore difficult to identify exact causes for the lack of bubbles. However, some factors, such as individual susceptibility to bubble formation may be causative. In previous studies, where an identical dive profile has been used, bubble formation was dependent on body weight, and rats weighing more than 300 g generally produced considerably more bubbles than rats weighing less than 300 g (Wisloff et al. 2003). Obesity is a well-known risk factor for DCS, due to greater solubility of nitrogen in fat compared to other tissues. Adipose tissue also has a low perfusion rate, causing nitrogen to be eliminated at a slower rate (Francis and Mitchell, 2003a). The mean weight of the animals in the present study was 280 g. Thus, it is possible that weight was a limiting factor with regard to bubble formation. However, the sample sizes were relatively small in both the present study and the study by Havnes et al. (2010) (Havnes et al. 2010). Furthermore, there is insufficient knowledge of S100B and NSE expression, and on the cascade from decompression to the symptomatology of DCS. Thus, there is need for caution when inferring the causes of the lack of S100B and NSE expression in the present study.

## Conclusion

The present study examined whether the protein biomarkers S100B and NSE could be found in serum from rats after exposure to a simulated dive to 700 kPa for 45 min breathing air. There were no differences in serum concentrations before versus after the dive exposure. This may be explained by the lack of vascular gas bubbles after the dives.

## Conflict of Interest

The authors report no conflict of interest. The authors alone are responsible for the content and writing the paper.
